# Biosynthesis of Zinc Oxide Nanoparticles Using *Bacillus paramycoides* for In Vitro Biological Activities and In Vivo Assessment Against Hepatorenal Injury Induced by CCl_4_ in Rats

**DOI:** 10.1007/s12010-023-04817-y

**Published:** 2024-01-04

**Authors:** Heba A. El-Refai, Alaa M. Saleh, Shimaa I. A. Mohamed, Asmaa F. Aboul Naser, Rania A. Zaki, Sanaa K. Gomaa, Manal A. Hamed

**Affiliations:** 1 Chemistry of Natural and Microbial Products Department, National Research Centre, Dokki, Giza, Egypt; 2 Department of Therapeutic Chemistry, National Research Centre, Dokki, Giza, Egypt

**Keywords:** Zinc oxide nanoparticles, CCl_4_, Cytotoxicity, Antioxidant, Antibacterial, Hepatorenal injury

## Abstract

Recently, impressive developments in the field of nanotechnology have been achieved. The study aimed to synthetize zinc oxide nanoparticles (ZnONPs) from locally isolated terrestrial *Bacillus paramycoides* (MCCC 1A04098) bacteria and assess its role as antioxidant, antimicrobial, and anticancer agent. The antioxidant activity was done using the percentage of DPPH scavenging method. The antibacterial activity was evaluated against *Escherichia coli*, *Staphylococcus aureus*, *Bacillus cereus*, and *Candida albicans.* The anti-proliferation assay against hepatocellular carcinoma (HepG2) and human breast cancer (MCF-7) cell lines was estimated by neutral red assay. The apoptotic effect of ZnONP was measured by flow cytometry. The in vivo evaluation was carried out against hepatorenal injuries induced by carbon tetrachloride (CCl_4_) in rats comparing with silymarin as a reference drug. The oxidative stress markers, liver and kidney function enzyme indices, lipid profile, and the histological features of the liver and kidney were also examined. ZnONPs revealed antioxidant and antibacterial effects. It also exerted cytotoxic and apoptotic effect in a dose dependent manner without any toxicity on normal cell line. ZnONPs improved all the biochemical parameters under investigation to varying degrees, and the histological pictures of the liver and kidney confirmed the results. In conclusion, ZnONPs were successfully synthesized from the terrestrial *Bacillus paramycoides* and recorded in vitro antioxidant, anticancer, and antibacterial effects as well as in vivo anti-hepatorenal toxicity effects.

**Graphical Abstract:**

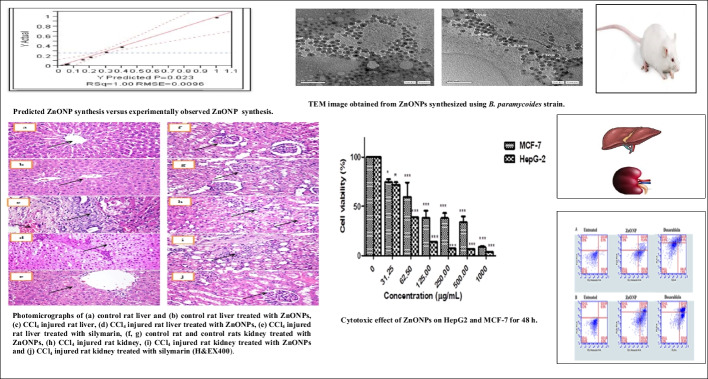

## Introduction

Microorganisms have emerged as a unique method for the manufacture of metal nanoparticles, which has the potential to be favored over existing chemical and physical processes. The use of nanotechnology in packaging materials is one example of how nanotechnology can be used in the food sector to control the microbial of growth of pathogenic bacteria [1]. In addition, nanomaterials are being studied for a variety of purposes, including microbial growth suppression [2] and as antibiotic carriers [3].

Zinc oxide shows antibacterial potential which is inversely proportional to particle size [4]. Zinc, zinc oxide, and zinc sulfide nanoparticles are used in a variety of biomedical applications due to their biological characteristics. The surface of ZnONPs is designed and modified for killing tumor cells and in drug delivery applications [5]. Nano zinc oxide is employed as a catalyst in traditional petrochemical industries, as well as for absorbing ammonia gas and removing H_2_S from drilling fluid [6].

At room temperature, 1–200 nm nanoparticles are produced using microorganisms in factories. The recovery of biological metals by metal ion reduction or metal sulfide formation has been made possible by microbial resistance to heavy metal ions. By decreasing these ions and producing water-soluble form complexes, the defensive mechanism is strengthened [7]. The microbially synthesized ZnONPs may have antimicrobial potential while its mechanism of action is still understudy. Smaller NPs may have higher surface reactivity and be easier to penetrate cells, releasing Zn^2+^, which is toxic to bacterial cell macromolecules and causes cellular death, according to the possible bactericidal processes of ZnONPs [8]. Another hypothesis is that the ZnONPs induced the oxidative stress that resulted in reactive oxygen species production and bacterial cell destruction [9]. Due to their positive zeta potential, ZnONPs may attach to negatively charged bacterial cell leading to their accumulation and penetration into bacterial cell that lead to bacterial cell death [10].

According to several studies, different morphologies (particle size and shape) of ZnO show varying degrees of antibacterial activity [11]. Increased amounts of reactive oxygen species (ROS), primarily hydroxyl radicals, H_2_O_2_, and single oxygen are produced by aqueous suspensions of ZnO and contributing to the antibacterial action of ZnONPs [12]. According to Zhang et al. [13], chemical interactions between H_2_O_2_ and bacteria are the most important mechanism for antibacterial activity, where ZnONP’s surface abrasiveness has been shown to cause *E*. *coli* cell wall disruption [14].

Several studies revealed that nanoparticles have the capacity to combat and protect against cancer disease [15]. Cancer treatment regimens mainly chemotherapy, radiotherapy, and immune therapy possess severe side effects and insignificant outcomes. Recently, nanostructures mainly metal oxides gain a great awareness from scientists for cancer treatment due to their low cost and high efficiency [16].

The liver and kidney are the main target organs affected by xenobiotics [17, 18]. One of the most common toxins used in the induction of organ experimental toxicity is carbon tetrachloride (CCl_4_). CCl_4_ is a chemical compound that promotes the process of oxidative stress and cell destruction by two separate processes which are the covalent binding to protein membranes and the enhancement of lipid peroxidation that causes tissue damage [19].

Treatments by natural products rich in antioxidants attracted many researchers to focus their works on its safety and efficacy for treating many diseases [17–19]. Silymarin is a naturally occurring polyphenolic compound isolated from *Silybum marianum* and known to have antioxidant, anti-inflammatory, and antifibrotic properties [17–19]. Silymarin recorded a strong therapeutic effect against liver inflammation due to its richness with flavonolignans that protect the liver against peroxidation of lipids and depreciation of the antioxidant status via decreasing the level of nitric oxide, superoxide anion production, and glutathione concentration [17, 19]. It also exerts anti-renal toxicity by decreasing the renal oxidative damage and preserving the renal function and the histopathological architectures [18].

Because ZnONPs have antidiabetic, anti-inflammatory, and antioxidant effects [20], therefore, the aim of this study was to produce ZnONPs by a locally isolated bacterial strain (*Bacillus paramycoides*; MCCC 1A04098), optimizing the physiological circumstances for the nanoparticle formation and studying its characterization. The work was extended to investigate its in vitro therapeutic applications as an antibacterial and anticancer agent and its in vivo role as an anti-hepatorenal injury compared with silymarin as a reference drug.

## Materials and Methods

All methods were carried out in accordance with relevant guidelines and regulations. All experimental protocols were approved by the Projects Research Committee at National Research Centre, Cairo, Egypt (Approval number: 12020210, 2019–2022).

### Chemicals

All utilized components of media were of the highest purity and obtained from Oxoid (UK), Difco (USA), Merck (Germany), and Sigma-Aldrich (St Louis, MO, USA).

### Bacterial Screening for ZnONPs Biosynthesis and Characterization

Nutrient broth (100 ml) was used for culturing bacterial isolates in a 250-ml Erlenmeyer flask that was then incubated in a rotatory shaker incubator at 30 °C for 48 h. Cultures were then centrifuged by cooling centrifuge at 4 °C and 10,000 rpm to obtain the supernatant for the synthesis of ZnONPs. The supernatant (20 ml) was then mixed with equal volume of 4 mM ZnSO_4_, 0.2% P.V.P (Polyvinyl pyrrolidone), and 0.2% isopropanol then exposed to a dose of 15 kGy of gamma radiation applied at National Center for Radiation Research and Technology (NCRRT), Cairo, Egypt. Formation of ZnONPs was detected by visual observation of color change. Then, further confirmation by UV–Visible spectrophotometer (JASCO V-560) at a range of 200–800 nm was done [21], and the culture filtrate was used as a control under the same experimental conditions.

The obtained pellet was used for characterization via FTIR, XRD (XRD 6000 series, Shimadzu, Tokyo, Japan), and TEM (JEOL electron microscopy JEM-100 CX). Additionally, the dynamic light scattering (DLS) was carried out to record the stability of ZnO nanoparticles; this was checked by zeta potential measurement using a Malvern Zetasizer Nano ZS (Malvern Instruments Ltd., UK).

### Optimization of ZnONPs Using Plackett Burman Design

The most important factors regarding the synthesis of ZnONPs were clarified via a screening experiment [22]. This design is suggested when more than five factors are under investigation [23]. Seven factors (X_1_, salt concentration (mM); X_2_, filtrate concentration (ml); X_3_, PVP (%); X_4_, isopropanol (%); X_5_, time (h.); X_6_, temperature (°C); and X_7_, radiation (KGy)) were evaluated in nine combinations organized according to the statistical design.

All trials were performed thrice, and the response was calculated as the average dry weight of the synthesized ZnONPs. Each variable main effect was calculated as the variance between the average of measurements at the high setting ( +) and the average of measurements at the low setting ( −) of that factor. To determine the significance of the variable, *t-*values and *p-*values were calculated. ANOVA was adopted for the determination of the significance of the model, and the regression equation was also gotten. Values that represented the level of *p* < 0.05 were considered to be significant.

### In Vitro Antioxidant Activity

For DPPH free radical inhibition activity, a 100 µl sample (concentration 100, 250, 500, 1000 µg ZnONPs/ml deionized water) was mixed with 900 µl of 0.1 mM DPPH solution in methanol. The mixture was shaken vigorously and left to reach a steady state in the dark for 30 min at a temperature of 37 °C [24]. DPPH decolorization was determined by measuring the absorbance at 517 nm, and the DPPH radical scavenging effect was calculated according to the following equation:

$$\mathrm{Inhibition\;}\left(\mathrm{\%}\right)=\left({{\text{A}}}_{1}-{{\text{A}}}_{2}/{{\text{A}}}_{1}\right)\times 100$$

where A_1_ was the absorbance of the DPPH solution without the sample and A_2_ was the absorbance of DPPH with the sample. Ascorbic acid was used as a standard, and all the tests were performed in triplicate.

#### In Vitro Cytotoxicity Assay

Cytotoxicity of ZnONPs on different cancer cell lines, mainly hepatocellular carcinoma (HepG-2) and human breast cancer (MCF-7) as well as normal cell human skin fibroblast (HSF), was evaluated by neutral red assay. All cell lines were purchased from ATTC (American Type Culture Collection). Cells were cultured in complete culture media of Dulbecco’s Modified Eagle’s Medium (DMEM) (Lonza, Belgium) supplemented with 10% heat-inactivated fetal bovine serum, 100 mg/mL streptomycin and 100 units/mL penicillin. The cells were incubated and maintained at 37 °C in a humidified incubator with a 5% (v/v) CO_2_ atmosphere. Aliquots of 200 µL of cell suspension (5000–20,000) were seeded in 96-well flat-bottom plates and allowed to attach overnight. After 24 h, the cells were treated with the ZnO nanoparticles at different concentrations (0, 32.25, 62.50, 125, 250, 500, 1000 µl/mL) for 48 h. After the incubation time, the cells were washed with 150 µl of Dulbecco’s phosphate-buffered saline (DPBS). A total of 100 µl neutral red solution (0.4 µg/ml) (Sigma-Aldrich) was added to each well, then incubated for 2 h at 37 °C and 5% CO_2_. Finally, 150 µl of de-stain solution for neutral red (1% acetic acid: 50% ethanol (96%): 49% deionized H_2_O) was added, and then the plate was shacked rapidly for at least 10 min on a micrometer plate shaker before the measurement of color intensity at 540 nm in a microtiter plate reader spectrophotometer (Sorin, Biomedica S.p.A., Milan, Italy), using blanks which contain no cells as a reference. Doxorubicin (Dox, Mr = 543.5) was used as a positive control. Dimethyl sulfoxide (DMSO) was used as a vehicle to dissolve the tested extract.

The percentage of cell viability was calculated according to the following equation:

$$\mathrm{Cell\;viability\;}\left(\mathrm{\%}\right)=\mathrm{OD\;Treatment}-\mathrm{OD\;blank}/\mathrm{OD\;control}-\mathrm{OD\;blank}*100.$$

Each experiment group was repeated three times.

### Determination of Cell Death—Annexin V/Propidium Iodide Double Staining

The capacity of the tested nanoparticle to induce programmed cell death (apoptosis) was measured using Annexin V-FITC apoptosis detection kit (Abcam Inc., Cambridge Science Park, Cambridge, UK) coupled with 2 fluorescent channels flow cytometry. Cells were treated with ZnONPs and doxorubicin (10 µM) for 48 h as positive control. Cell suspension with a density (1 × 10^5^ cells) was collected and washed twice with ice-cold PBS (pH 7.4). According to the manufacturer’s protocol, the collected cells were stained with 5 µL of Annexin V-FITC/PI solution for 30 min at room temperature in the dark. After labeling, cells were injected using an ACEA NovocyteTM flow-cytometer (ACEA Biosciences Inc., San Diego, CA, USA), and the fluorescent signals of FITC and PI were then detected using FL1 and FL2 signal detectors, respectively (ex/em 488/530 nm for FITC and ex/em 535/617 nm for PI) [25].

### Antimicrobial Activity of ZnONPs

The antimicrobial activity was carried out in nutrient agar plates according to Mostafa et al. [26]. The pathogenic microorganisms used in this study were Gram-positive bacteria *Bacillus cereus* (ATCC 6629), *Staphylococcus aureus* (ATCC 6538), Gram-negative bacteria *Escherichia coli* (ATCC 25922), and pathogenic yeast *Candida albicans* (ATCC 10231)*.*

Each microorganism was incubated for 24 h in nutrient broth at 37 °C from which inoculum was taken and adjusted to approximately 0.5 ml McFarland standard (1.5 × 10^8^ CFU /ml). Inoculum (25.0 µl) was inoculated into each petri dish containing 20 ml sterile nutrient agar and left to cool and solidify. A total of 100 µl of different concentrations of ZnONPs (10, 20, 30, 40 mg) suspended in 1 ml (DMSO) was applied into the 0.9-cm well of each inoculated agar plates which were prepared previously by using 1.0-cm cork borer applying Well Diffusion Method. Standard antimicrobial (chloramphenicol and nystatin) was used as reference at concentration (1 mg/1 ml), and DMSO was applied as control. Seeded plates were placed in the refrigerator for 1 h for more diffusion of these samples, followed by incubation at 37 °C for 24 h, and zones of inhibition (ZI) were measured in mm [26].

### In Vivo Evaluation

#### Animals and Ethics

In this study, male Wistar albino rats (120–140 g) were obtained from the animal house at the National Research Centre, Egypt. All animals were housed in standard plastic cages (10 rats per cage) in an environmentally controlled condition at a temperature of 25 ± 2 °C with free access to water and diet. They were kept for 2 weeks for acclimatization before starting the experiment. Anesthetic procedures and handling with animals complied with the guidelines of the Medical Research Ethical Committee at the National Research Centre in Egypt (approval no: 19305).

#### Doses and Route of Administration

An intraperitoneal (i.p.) injection of carbon tetrachloride suspended in olive oil (1:9 v/v) was administered twice weekly for 6 weeks [27]. ZnONPs were administered orally for 6 weeks at a dose of 5 mg/kg b.wt/day [28]. The reference drug, silymarin, was administered orally for 6 weeks at a dose of 100 mg/kg body weight/day [29].

#### Experimental Groups

Fifty male rats were divided into five groups (10 rats each), where group 1 served as normal healthy control rats received vehicle (i.p olive oil), group 2 served as control rats received ZnONPs, group 3 was injected with CCl_4_, group 4 was intoxicated with CCl_4_ followed by treatment of ZnONPs, and group 5 was intoxicated with CCl_4_ followed by silymarin treatment.

#### Sample Preparation

At the end of the experimental period, all animals were anesthetized by diethyl ether. The blood was drawn from the sub-lingual vein and centrifuged at 300 g for 10 min to separate the serum, and the separated serum was kept at − 80 °C for liver and kidney function tests as well as the lipid profile analysis. For the antioxidant determinations, liver tissue was homogenized in cold 0.9 N NaCl (1:10 w/v), centrifuged at 300 g for 10 min, and then the supernatant was stored at − 80 °C.

#### Biochemical Assays

Oxidative stress markers, glutathione (GSH) and malondialdehyde (MDA), were estimated calorimetrically by the methods of Moron et al. [30] and Buege and Aust [31], respectively. The total antioxidant capacity (TAC) was estimated by using biodiagnostic kits (Biogamma, Stanbio, West Germany). The lipid profile, total cholesterol (TC), high-density lipoprotein-cholesterol (HDL-C), low-density lipoprotein-cholesterol (LDL-C), and triglycerides (TG), were determined by the method of Meiattini et al. [32], Bustein et al. [33], Assmann et al. [34], and Fossati and Prencipe [35], respectively. Liver function enzymes, aspartate and alanine aminotransferases (AST and ALT), were measured by the method of Gella et al. [36], while alkaline phosphatase (ALP) was assayed by the method of Rosalki et al. [37]. Kidney function indices, urea and creatinine, were determined by the method of Tabacco et al. [38] and Bartels and Böhmer [39], respectively, while the albumin level was tested by using bio-diagnostic kits (Biogamma, Stanbio, West Germany).

### Histopathological Analysis

Samples of the liver and kidney were fixed in 10% formalin. For sectioning, paraffin-embedded samples were produced at a 4-µm thickness. Hematoxylin and eosin were used to stain the slides, and they were then inspected under a light microscope [40].

### Statistical Analysis and Calculations

All data of the in vivo study were expressed as mean ± SD of 10 rats in each group. Statistical analysis was carried out by one-way analysis of variance (ANOVA), Costat Software Computer Program accompanied by *post-hoc* test at least significance difference (LSD) between groups at *p* < 0.05. The percentage change versus control (− or +) and % of improvement were calculated according to Motawi et al. [41], where negative control was the normal healthy rats and positive control was the CCl_4_-induced rats.

$$\begin{array}{c}\mathrm{\%\;change}=\left\{\left(\mathrm{Mean\;of\;control\;group}-\mathrm{Mean\;of\;treated\;group}\right)/\mathrm{Mean\;of\;control\;group}\right\}\times 100\\ \mathrm{\%\;of\;improvement}-\left\{\left(\mathrm{Mean\;of\;}{{\text{CCl}}}_{4}-\mathrm{Mean\;of\;treated\;group}\right)/\mathrm{Mean\;of\;control\;group}\right\}\times 100\end{array}$$  

## Results and Discussion

### Bacterial Isolates Screening for ZnONPs Biosynthesis

For ZnONPs bio-production, the nutrient broth was used as inoculum media for bacterial strains and left for 2 days incubation at 30 °C. UV–Vis spectrophotometer was used for the optical absorption spectra of ZnONPs. Strong UV–Vis absorption peak at 395 nm indicated the formation of ZnONPs and broad peak designated polydisperse particles. The wavelength of surface plasmon absorption is affected by the particle size and form of metal nanoparticles [42].

The wavelength of absorbance and the peak width (non-displayed results) indicated that from all the screened bacterial strains, only the isolate from soil was capable to synthesize ZnONPs. This isolate was identified by Sigma Company of Scientific Services, Egypt (http://www.sigma-co-eg.com) as *Bacillus paramycoides* strain MCCC 1A04098; it was assigned the accession number MT102429A in the Gene Bank.

### Statistical Optimization of ZnONPs by Placket Burman Design

The reaction conditions have a significant effect on the ZnONPs synthesized by various bacterial strains [12]. Experimental studies were used to determine the upper and lower limits for the parameters. Placket Burman statistical design generated a nine-run table (Table 1). The significant impact for each parameter for influencing both ZnONPs size as well as dry mass (yield) of the nanoparticles was investigated (non-displayed results), where the percentage of PVP, the temperature of the reaction (°C), and the concentration of the filtrate (ml) had a positive influence on ZnONPs synthesis, whereas the other variables had a negative influence. The data in Table 1 showed that the particle diameter of ZnONPs ranged from 10.30 to 20 nm on average. The particle size ranged between 4.37 and 7.45 nm in trials 2 and 8, which could be attributed to the effect with both high salt concentration and temperature to high gamma radiation. Although at low temperature and no gamma radiation in trials 4, 5, and 6, the particle size was 17.30–20 nm.

**Table 1:** Optimization of ZnONPs production by Placket-Burman design

Trial no	Variables							Dry weight (mg)/ 50 ml	Average of particle size (nm)
	X_1_	X_2_	X_3_	X_4_	X_5_	X_6_	X_7_		
1	− (2)	− (0.50)	− (0.10)	+ (0.30)	+ (96)	+ (100)	− (non)	0.05	17.20
2	+ (6)	− (0.50)	− (0.10)	− (0.10)	− (48)	+ (100)	+ (35)	0.06	7.45
3	− (2)	+ (1.50)	− (0.10)	− (0.10)	− (48)	− (37)	+ (35)	0.40	14.30
4	+ (6)	+ (1.50)	− (0.10)	+ (0.30)	+ (96)	− (37)	− (non)	0.20	17.30
5	− (2)	− (0.50)	+ (0.30)	+ (0.30)	− (48)	− (37)	− (non)	0.20	20.00
6	+ (6)	− (0.50)	+ (0.30)	− (0.10)	+ (96)	− (37)	− (non)	0.15	20.00
7	+ (6)	+ (1.50)	+ (0.30)	− (0.10)	− (48)	+ (100)	− (non)	0.30	15.50
8	+ (6)	+ (1.50)	+ (0.30)	+ (0.30)	+ (96)	+ (100)	+ (35)	1.00	4.37
9	0(4)	0 (1)	0 (0.20)	0 (0.20)	0(48)	0 (50)	0(15)	0.03	10.30

**Fig. 1:**
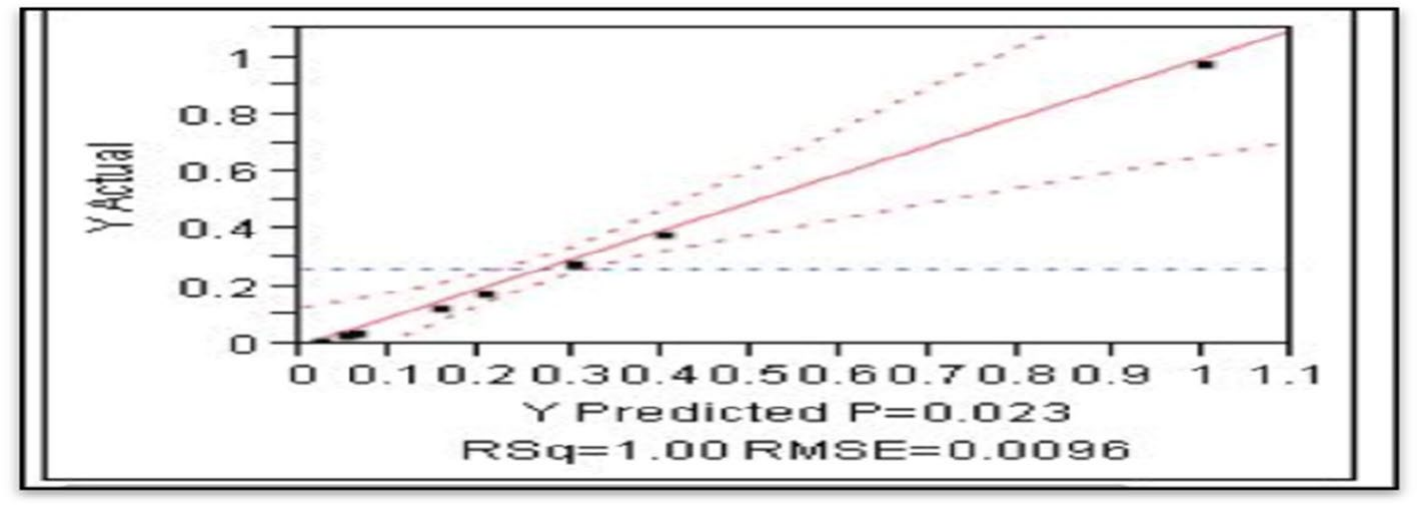
Predicted ZnONP synthesis versus experimentally observed ZnONP synthesis

Table 2 summarizes the ANOVA analysis. The actual by-predicted plot (Fig. 1) showed that the predicted *R*^2^ (0.96) was in acceptable agreement with the adjusted *R*^2^ (1.0), and the model’s *p*-value was significant (0.02). A verification experiment was performed in triplicate to determine the accuracy of this experiment (non-displayed results). The antibacterial activity of ZnONPs (trial 8) (4.37 nm) was higher, which was consistent with the findings of Siddiqi et al. [43] who stated that the antibacterial activity of MgO was dependent on particle size in vitro and established the bactericidal effect against Gram-positive and Gram-negative bacteria. In this research, we aimed to produce particles with a size of 4.37 nm, which strongly endorsed antibacterial potential. This was accomplished with a yield of 1.00 mg/50 ml under the production parameters that were estimated in trial 8.

**Table 2:** ANOVA analysis for ZnONPs synthesis

Term	Coefficient	S.E	*t*-value	*p*-value
Intercept	0.185	0.004	51.03	0.013*
Salt concentration (mM)	0.176	0.005	37.61	0.017*
Filtrate concentration (ml)	0.069	0.004	17.03	0.037*
PVP (%)	− 0.217	0.007	− 29.70	0.021*
Isopropanol (%)	0.186	0.005	39.74	0.016*
Time (h)	− 0.015	0.005	− 3.12	0.197
Temperature (°C)	0.168	0.004	44.04	0.015*
Radiation (K Gray)	0.446	0.009	51.76	0.012*

### Characterization of ZnONPs

**Fig. 2:**
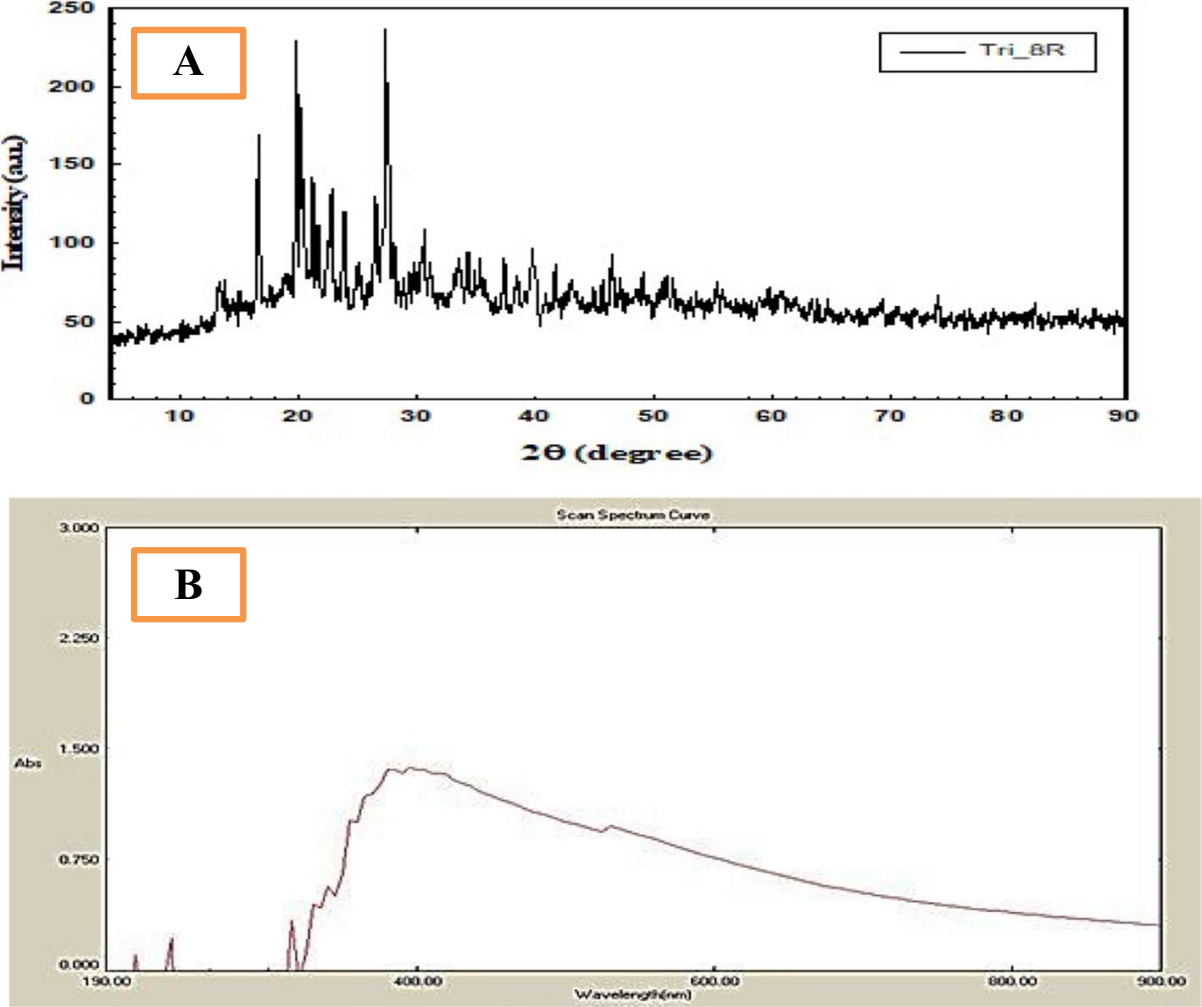
**A** UV–*vis* spectrum and **B** XRD of ZnONPs

In this study, the color of the bacterial extract was changed to turbid white after adding zinc sulfate as a source for ZnONPs. Figure 2A indicated that the UV–Vis spectra of the bacterial synthesized ZnONPs confirmed a significant peak of 395 nm, which is mostly distinguished by ZnONPs. These results were in accordance with Mohamed et al. [44] who stated that the surface plasmon resonance (SPR) of green synthesized ZnONPs was between 370 and 400 nm**.**

XRD was used for detecting the crystallinity of ZnONPs which elucidated the atoms’ states, axes, and sizes. According to the results in Fig. 2B, pure ZnO particles exhibit different diffraction peaks in the (100), (002), (101), (102), (110), (103), and (112) planes, which are represented by the XRD patterns of ZnONPs powder. The collective diffraction peaks support crystalline ZnO with a hexagonal wurtzite structure, which is consistent with the findings of Zhong et al. [45].

**Fig. 3:**
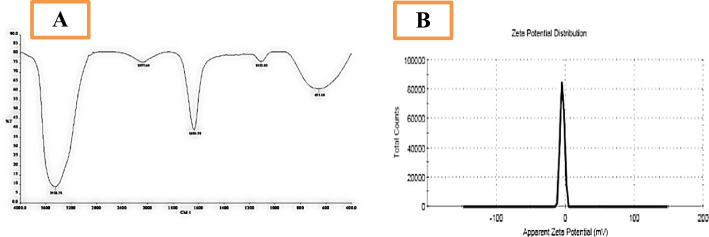
**A** FTIR spectrum and **B** zeta potential curve of ZnONPs

The existence or absenteeism of certain functional groups was confirmed by FTIR analysis. FTIR spectrum of ZnONPs obtained in T8 (Fig. 3A) showed strong and better resolved vibrational absorption with bands of 3452.71, 2077.64, 1634.79, 1112.81, and 655.10 bands of 3452.71 that indicated the presence of hydroxyl group (O–H), bands of 2077.64 indicated the occurrence of C = C stretching vibration, and bands of 1634.79 indicated the presence of C = O symmetric stretching. The peaks at 1112.81 cm^−1^ may be attributed to –C–O and –C–O–C stretching modes. The peak in the region between 400 and 600 cm^−1^ is allotted to Zn–O [46].

The amplitude of the zeta potential indicates the colloid stability and the surface charge characteristics of these nanoparticles. Once particles are in the suspension with large either negative or positive zeta potential values, the particles repel from one another or there is no agglomeration of nanoparticles. In contrast, Devi and Velu [46] stated that there is no force to keep particles from getting together and aggregating if their zeta potential values are low. In the present results and in agreement with [46], the zeta potential measurement was determined to be − 4.41 mV in Fig. 3B, indicating that ZnO nanoparticles are more stable in suspension and have an antibacterial effect.

**Fig. 4:**
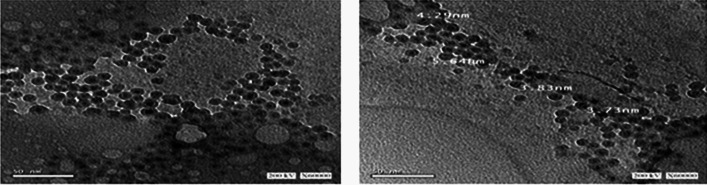
TEM image obtained from synthesized ZnONPs using *Bacillus paramycoides* strain MCCC 1A04098

Synthesized ZnONPs were characterized using TEM (Fig. 4). The findings showed that in trial 8, ZnONPs had a hexagonal form with a size range of 3.74 to 5.64 nm, and the mean of the average size was 4.37 nm. In experiment 8, ZnONP’s size and shape were uniform.

### Free Radical Scavenging Potential

The scavenging potential of the microbially obtained zinc oxide nanoparticles was found to be 35 ± 0.60% at a concentration of 1000 µg/ml. The IC_50_ of biogenic ZnONPs was 2820 µg/ml by comparison with ascorbic acid (21 µg/ml) (Table 3). In line with our results, it was found that the green synthesized ZnONPs either from plants or microbial origin had scavenging activity that may be attributed to its capping with biological contents that possess free radical scavenging activity [47, 48].

**Table 3:** DPPH^−^ scavenging activity of ZnONPs

Sample	Concentration (µg/mL)	DPPH^−^ (% inhibition)
ZnONPs		
	100	27 ± 0.85
	250	29.5 ± 0.42
	500	30.3 ± 0.87
	1000	35 ± 0.60
	IC50	2820.00
Ascorbic acid		
	15	50.1 ± 1.46
	25	80.43 ± 0.94
	50	91.4 ± 0.83
	100	97.36 ± 0.74
	IC50	21.00

Data are mean ± SE at *n* = 3

### Antimicrobial Activity

Biosynthesized ZnONPs have been always tested against human pathogens for their antimicrobial activity [49]. In this study, the agar well diffusion method was adopted for testing the antimicrobial activity of ZnONPs and compared to standard antibiotics. The zone of inhibition increased with the increase in ZnONPs concentration. Maximum activity was observed against *E*. *coli* where the inhibition zone was 30 mm at a concentration 40 mg/ml DMSO, while chloramphenicol was 32 mm at a concentration of 1 mg/1 ml (Table 4). According to Balraj et al. [50], the well-diffusion test for antimicrobial activity of biologically synthesized ZnONPs showed a wide inhibition zone against *E*. *coli* than *B*. *subtilis*. This observation may be owing to the different constitutions of the cell walls of gram-negative and gram-positive bacteria [12]. Earlier reports had suggested that the antimicrobial effect of ZnONPs may be a result of either the induction of excess reactive oxygen species or the initiation of Zn^2+^ release leading to microbial cell damage [51]. Although ZnO particles in the nano-scale show promising antimicrobial activity, the exact mechanism has not been well established [52].

**Table 4:** Inhibition zone diameter (millimeter) of the ZnONPs

Test organisms	10 mg/ml	20 mg/ml	30 mg/ml	40 mg/ml	Standard Ab	DMSO
*E*. *coli*	23	25	28	30	32	Nil
*S*. *aureus*	19	20	27	29	18	Nil
*B*. *cereus*	14	16	19	21	12	Nil
*C*. *albicans*	13	17	19	20	30	Nil

Nil, no antimicrobial activity recorded

### Cell Viability Assay

**Fig. 5:**
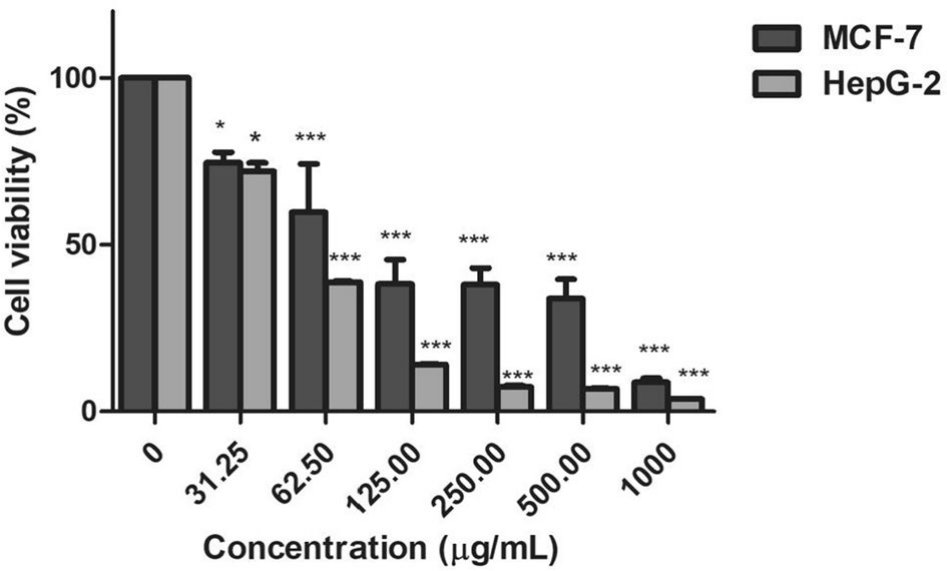
Cytotoxic effect of ZnONPs on HepG2 and MCF-7 for 48 h at different concentrations (from 31.25 up to 1000 µg/mL). Data are demonstrated as a mean ± SD of three identical experiments made in three replicates. *p < 0.01; **p < 0.001, ***p < 0.0001 by one-way ANOVA and Dunnett’s test

Neutral red assay was performed for cytotoxicity testing to measure the cytotoxicity of ZnONPs toward human adenocarcinoma breast cancer (MCF-7) cell line and liver (HepG-2) cancer cell line at several concentrations (31.25–1000 µg/mL) for 48 h. The information showed that ZnONPs had a concentration-dependent inhibitory effect on the viability of cells from the MCF-7 and HepG-2 cell lines (Fig. 5). The IC_50_ values (the half maximal inhibitory concentration) were calculated for ZnONPs and the positive control doxorubicin (non-displayed results). ZnONPs exhibited significant cytotoxic activity on HepG-2 with IC_50_ value of 57.47 µg/mL and the percentage of cell viability inhibition reached up to 96% at a concentration of 1000 µg/ml. For MCF-7 cell proliferation, ZnONPs inhibited up to 85%. IC_50_ of ZnONPs was determined (169.35 µg/ml) after 48 h, and it was reported that the nanoparticles’ physical features, such as shape, size, and chemical composition and concentrations, are able to control the growth of the cancer cells [53]. The size of nanoparticles is very tiny that enables them to penetrate the cells easily and cause cellular damage as well as their ability to produce destructive reactive oxygen species that are responsible for their cytotoxic activities [54]. The results indicated that ZnONPs exhibited no cytotoxicity on HSF normal cell line. Therefore, the nanoparticle had the ability to distinguish between cancerous and normal cell lines.

### ZnONPs Induce Programmed Cell Death

**Fig. 6:**
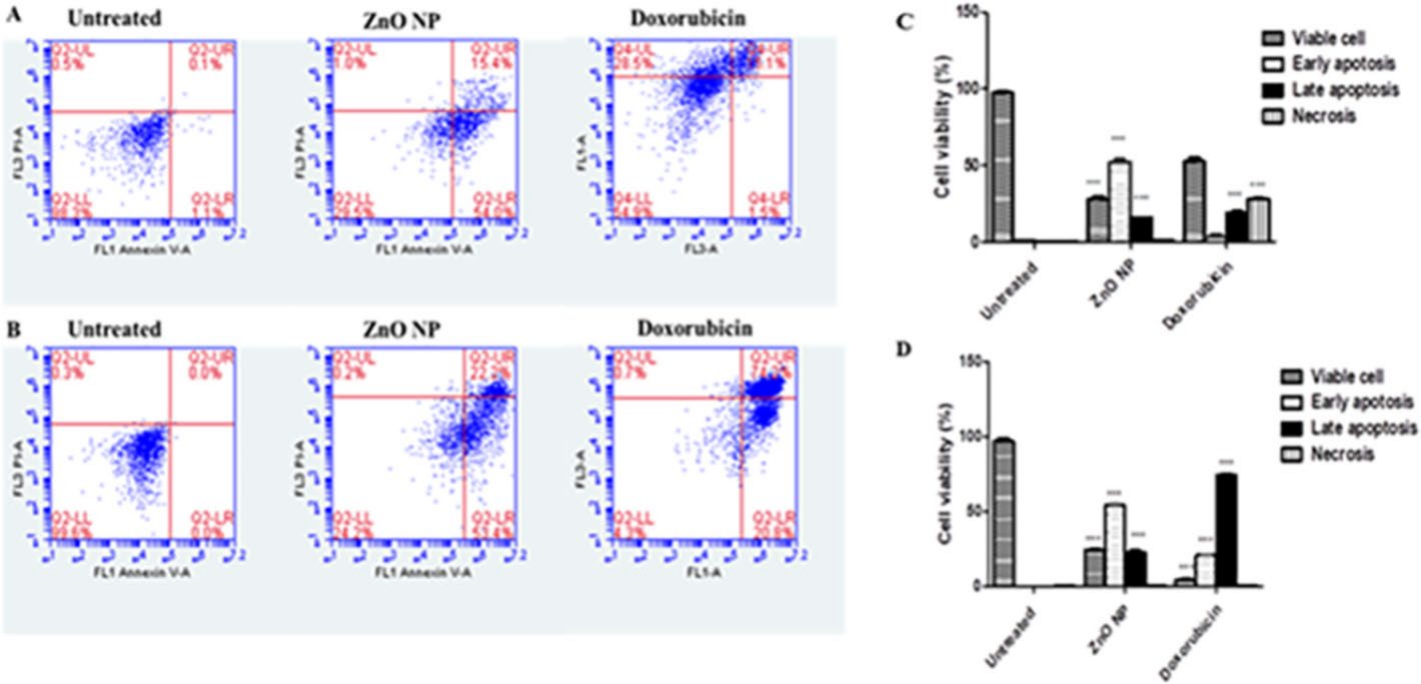
Representative FACS analyses of Annexin V and PI staining. **A** HepG2 and **B** MCF-7 cells treated with ZnONPs for 48 h. Flow cytometric analysis of the Annexin V and PI apoptotic assay of **C** HepG-2 and **D** MCF-7 cells exposed to ZnONPs for 48 h. Each histogram represents the mean ± SD from three independent experiments. *p < 0.05 and **p < 0.01, ***p < 0.001 compared with the control as determined by one-way ANOVA

The potential of ZnONPs to induce cell death via apoptosis rather than necrosis was evaluated, where HepG-2 was pre-treated using IC_50_ values of ZnONPs (57.47 µg/ml), and doxorubicin as a positive control was pretreated at 0.60 µg/ml. The percentage of early and late apoptosis was measured using a dual staining kit (Annexin V /PI) following 48 h of treatment. In ZnONPs treated cells, the percentage of living cells decreased after treatment for 48 h from 96.61 to 29.50%. The percentage of early apoptosis was 54% while the percentage of late apoptosis was 15.40%. This indicated that ZnONPs induced cell death via apoptosis rather than necrosis. Doxorubicin showed an apoptotic effect and necrosis by 28.50% (Fig. 6A and C). On the other hand, MCF-7 was treated with the IC_50_ values of ZnONPs at 169.35 µg/ml and doxorubicin as a positive control at concentration of 1.20 µg/ml. These results indicated that ZnONPs showed an apoptotic effect on MCF-7 cell line. The percentage of viable cells in ZnONPs-treated cells showed a significant decrease from 99.60% of untreated cells to 24.20%. Furthermore, the percentages of early and late apoptosis were 53.40 and 22.20%, respectively (Fig. 6B and D). The results indicated that ZnONPs induced programmed cell death (apoptosis) in treated cell lines rather than necrosis. Doxorubicin was used as positive control at 1.20 µg/ml, and the percentage of viable cells was decreased to 4.2% with a marked increase in the percentage of late apoptosis (72.20%) and early apoptosis up to 20.80%. It was reported that the NPs have the capacity to enter the small pores of the cells. The prepared NPs are capable of entering cells (∼20 µm) effortlessly due to their extremely small size (∼4.37 nm) in comparison with the cells [55, 56]. Quick formation of agglomerates occurs due to small NPs high density in liquid system, and it is presumed that these agglomerates of ZnONPs disrupt cell organelles such as endoplasmic reticulum, DNA, RNA, and mitochondria [57]. Furthermore, oxidative stress can be used to promote programmed cell death by producing ROS that enter the cell’s outer wall and subsequently infiltrate the membrane’s inner wall. These ROS interact with cellular organelles, causing enzymatic alterations and disturbance of cellular contents [58].

### Oxidative Stress Markers

The antioxidants are considered as possible protecting agents that reduce oxidative damage of the human body from ROS and retard the progress of many diseases [59]. In the present study, rats given CCl_4_ showed much lower levels of TAC and GSH than the control group, but MDA levels significantly increased. GSH is a strong endogenous antioxidant through its ability to bind to ROS, thereafter maintaining its balanced level is critical for cell survival [60]. These outcomes were consistent with the findings of Morsy et al. [17]. TAC and GSH levels were significantly high in CCl_4_ rats treated with ZnONPs comparing with the CCl_4_ group, but MDA levels were much lower (Table 5). Therefore, TAC was improved by 33.81 and 36.66% upon treatment with ZnONPs and silymarin, respectively. GSH improved by 23.55 and 38.22%, respectively, whereas MDA improved by 69.01 and 89.89%, respectively. Zinc may have an influence on the control of cellular glutathione, which is important for cellular defense against ROS according to Bashandy et al. [20]. The same authors added that zinc is an essential component of superoxide dismutase enzyme, which regulates free radicals and oxidative stress. These results confirmed the observed amelioration effect of the oxidative stress markers after treatment with ZnONPs. In addition, we observed amelioration in the antioxidant indices after silymarin treatment due to its richness with flavonoids that attenuated the lipid peroxidation, nitric oxide, superoxide dismutase, and enhancement of glutathione level [17, 19].

**Table 5:** Effects of ZnONPs on oxidative stress markers and lipid profile in different studied groups

Groups	Oxidative stress markers			Lipid profile			
	GSH (µg/gm tissue)	MDA (µmol/mg protein)	TAC (mmol/l)	Cholesterol (mg/dl)	TG (mg/dl)	HDL (mg/dl)	LDL (mg/dl)
Control	16.22^a^ ± 2.66 --	4.55^c^ ± 0.50 **--**	2.10^a^ ± 0.15 --	85.90^c^ ± 8.50 --	70.33^c^ ± 7.50 **--**	144.50^a^ ± 12.66 **--**	65.80^c^ ± 6.11 **--**
ZnONPs	15.31^a^ ± 1.18 (− 5.61)	3.91^c^ ± 0.98 (− 14.06)	2.15^a^ ± 0.12 (+ 2.38)	83.13^c^ ± 7.90 (− 3.22)	73.80^c^ ± 6.95 (+ 4.39)	143.17^a^ ± 10.53 (− 1.00)	63.91^c^ ± 5.60 (− 2.87)
CCl_4_	6.33^c^ ± 1.72 (− 60.97)	10.50^a^ ± 1.13 (+ 130.70)	0.95^c^ ± 0.10 (− 54.76)	283.50^a^ ± 23.98 (+ 230.03)	260.66^a^ ± 21.65 (+ 270.62)	68.00^c^ ± 5.90 (− 50.94)	230.10^a^ ± 18.61 (+ 249.70)
CCl_4_ + ZnONPs	10.15^b^ ± 1.60 [+ 60.35]	7.36^b^ ± 1.50 [− 29.90]	1.66^b^ ± 0.13 [+ 74.74]	230.78^b^ ± 19.21 [− 18.60]	188.70^b^ ± 15.12 [− 27.60]	97.40^b^ ± 7.88 [+ 100.43]	118.13^b^ ± 9.30 [− 48.66]
CCl_4_ + Silymarin	12.53^b^ ± 1.70 [+ 97.95]	6.41^b^ ± 0.64 [− 38.95]	1.72^b^ ± 0.11 [+ 81.05]	213.10^b^ ± 14.03 [− 24.38]	160.23^b^ ± 14.11 [− 38.52]	110.88^b^ ± 9.51 [+ 63.10]	106.23^b^ ± 9.71 [− 53.83]

Data are presented as the mean ± SD of ten rats in each of the groups. Groups with the same letters are not significantly different at *p* < 0.05, whereas groups with different letters are significantly different. Values between packets are the percentage changes compared to the control group, while values between parentheses are the percentage changes versus the CCl_4_ group

### Lipid Profile Markers

After normal rats were given ZnONPs, no significant alterations in lipid profile indicators were seen. In comparison with the control group, rats injected with CCl_4_ showed a significant increase in cholesterol, TG, and LDL-C levels. However, a significant drop in HDL-C level was noticed. CCl_4_-induced rats treated with ZnONPs recorded a significant drop in cholesterol, TG, and LDL-C levels as compared with the CCl_4_ group, while a significant increase in HDL-C level was recorded (Table 5). Our findings were consistent with those of Morsy et al. [17], who found that injecting CCl_4_ increased TG, TC, and LDL-C levels while decreasing HDL-C levels. Therefore, protein synthesis suppression and phospholipid metabolism disruption may lead to alterations in lipoprotein levels [17]. The same authors added that these alterations in the lipids profile were reversed by treatment with silymarin. Treatments with ZnONPs and silymarin showed improvement in cholesterol by 61.40 and 82.00%, respectively. TG was improved by 102.32 and 142.79%, respectively, and LDL was improved by 170.16 and 188.25%, respectively, while HDL was improved by 20.35 and 29.67%, respectively.

### Liver Function Enzymes

In terms of liver function enzymes, after administering ZnONPs to normal rats, there were no significant changes in AST, ALT, and ALP levels. As opposed to the control group, rats given CCl_4_ had significantly higher levels of AST, ALT, and ALP. According to Tochukwu [61], CCl_4_ caused necrosis and apoptosis in injured hepatocytes that lead to cytosolic liver enzyme flow to the bloodstream. This occurred in tandem with serum ALT, AST, and ALP enzyme activity increment. Furthermore, James et al. [62] suggested that the increase in ALT and AST after CCl_4_ treatment could be due to hepatic cell membrane disruption and mitochondrial injury. CCl_4_-induced rats treated with ZnONPs recorded an obvious drop in AST, ALT, and ALP levels in comparison with the CCl_4_ group (Table 6). Amelioration in AST level by 59.03 and 77.99% were observed after treatment with ZnONPs and silymarin, respectively. ALT was enhanced by 62.32 and 81.65%, respectively, while ALP was enhanced by 48.44 and 57.86%, respectively. The observed improvement in liver function enzymes was due to the antioxidant effect of ZnONPs and silymarin that can defend the integrity of cell membrane against the oxidative stress damage [17, 19, 20].

**Table 6:** Effects of ZnONPs on liver and kidney function indices in different studied groups

Groups	Liver function enzymes			Kidney function indices		
	AST (unit/L)	ALT (unit/L)	ALP (unit/L)	Urea (mg/dl)	Creatinine (mg/dl)	Albumin (mg/dl)
Control	69.88^c^ ± 6.10 --	40.66^c^ ± 3.15 --	91.51^c^ ± 8.10 --	29.32.00^c^ ± 3.63 --	0.89^c^ ± 0.02 --	3.50^c^ ± 0.14 **--**
ZnONPs	71.30^c^ ± 6.50 (+ 2.03)	41.91^c^ ± 2.03 (+ 3.07)	89.73^c^ ± 7.12 (− 1.95)	30.30^c^ ± 2.91 (+ 3.34)	0.99^c^ ± 0.03 (+ 11.24)	3.66^c^ ± 0.50 (+ 4.57)
CCl_4_	140.50^a^ ± 9.13 (+ 101.06)	110.50^a^ ± 8.50 (+ 171.76)	165.25^a^ ± 10.30 (+ 80.58)	98.17^a^ ± 6.50 (+ 204.12)	2.95^a^ ± 0.50 (+ 231.46)	9.13^a^ ± 3.14 (+ 160.85)
CCl_4_ + ZnONPs	99.25^b^ ± 7.60 [− 29.36]	85.16^b^ ± 5.13 [− 22.93]	120.92^b^ ± 7.30 [− 26.83]	44.43^b^ ± 5.60 [− 50.17]	1.40^b^ ± 0.13 [− 52.54]	5.90^b^ ± 1.10 [− 35.37]
CCl_4_ + Silymarin	86.00^b^ ± 4.70 [− 38.79]	77.30^b^ ± 2.66 [− 30.05]	112.30^c^ ± 6.73 [− 32.04]	39.60^b^ ± 3.75 [− 55.60]	1.13^c^ ± 0.11 [− 61.70]	5.10^b^ ± 1.93 [− 44.14]

Data are presented as the mean ± SD of ten rats in each of the groups. Groups with the same letters are not significantly different at *p* < 0.05, whereas groups with different letters are significantly different. Values between packets are the percentage changes compared to the control group, while values between parentheses are the percentage changes versus the CCl_4_ group

### Kidney Function Indices

In terms of kidney function parameters, the reported changes in urea, creatinine, and albumin levels following ZnONP delivery to normal rats were minimal. Rats administered with CCl_4_ demonstrated an impressive increase in their levels as compared with the control group. Aziz et al. [18] showed that CCl_4_ recorded a significant increase in serum creatinine and uric acid. They attributed this effect to the elevation of oxidative stress that causes a decrease in glomerular filtration rate and leads to an increase in serum urea and uric acid levels. The antioxidant action of ZnONPs attenuated urea, creatinine, and albumin levels in CCl_4_-treated groups (Table 6). Treatments with ZnONPs and silymarin showed a decrease in urea levels by 152.60 and 169.01%, respectively, while creatinine levels were reduced by 174.16 and 204.50%, respectively. Furthermore, albumin levels were improved by 92.29 and 115.14%, respectively. Aziz et al. [18] added that silymarin could reduce the symptoms of kidney damage, protect renal function, and preserve kidney architecture through reducing renal oxidative damage.

### Histopathological Analysis

**Fig. 7:**
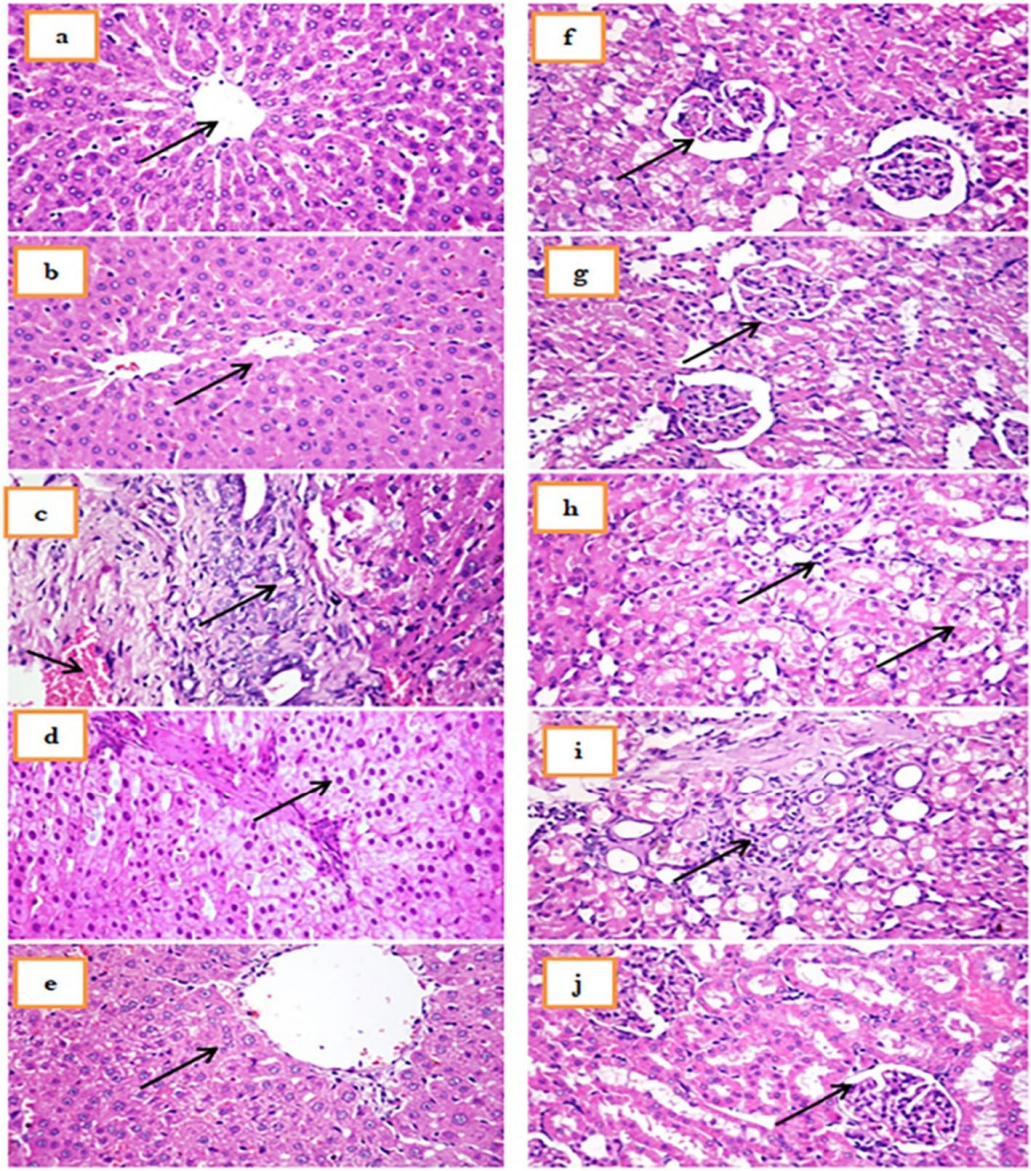
Photomicrographs of **a** control rat liver and **b** control rat liver treated with ZnONPs showing normal histological structure of central vein and surrounding hepatocytes, **c** CCl_4_-injured rat liver showing severe fibrosis of portal area with congestion of portal blood vessels, **d** CCl_4_-injured rat liver treated with ZnONPs showing vacuolation of hepatocytes, **e** CCl_4_-injured rat liver treated with silymarin showing mild vacuolar degeneration of hepatocytes, **f**, **g** control rat and control rat kidney treated with ZnONPs showing normal histological structure of renal corpuscles and renal tubules, **h** CCl_4_-injured rat kidney showing vacuolar degeneration and necrosis of renal tubular lining epithelium with karyocytomegaly, **i** CCl_4_-injured rat kidney treated with ZnONPs showing mononuclear inflammatory cells infiltration, and **j** CCl_4_-injured rat kidney treated with silymarin showing normal histological structure of renal corpuscles and renal tubules (H&EX400)

The liver in control rats together with those treated with ZnONPs displayed typical histological architectures of the central vein and its surrounding hepatocytes. In CCl_4_ group, and in agreement with the results of Morsy et al. [17], the liver showed bridging fibrosis, severe vacuolar degeneration of hepatocytes, portal blood vessel congestion, and bile duct hyperplasia. In CCl_4_ rats treated with ZnONPs, the liver showed mild vacuolation of hepatocytes. While in sylimarin-treated group, the rat liver displayed slight hepatocyte vacuolar degeneration (Fig. 7a–e). Bashandy et al. [20] hypothesized that zinc could protect the liver from fibrosis by reducing transforming growth factor-beta (TGF-β) that induced epithelial differentiation and fibroblast activation (the two prominent hallmarks of tissue fibrosis). Additionally, ZnONPs may reach to certain cells and structures, preserve their functions, and prevent death through ions exchange.

The kidney in control rats and in control rats treated with ZnONPs displayed normal histological structure of renal corpuscles and renal tubules. The kidney in CCl_4_-injured group showed severe interstitial fibrosis, vacuolar degeneration, and necrosis of renal tubular lining epithelium with karyocytomegaly. In CCl_4_ group treated with ZnONPs, the kidney showed mononuclear inflammatory cell infiltration, while in sylimarin-treated group, the rat kidney displayed normal renal corpuscles and renal tubule histological structure (Fig. 6f–j). Bashandy et al. [20] suggested the role of ZnONPs as anti-inflammatory and antioxidant agent and thus may protect renal damage via reducing the levels of IL-6 and lipid peroxidation process. Also, silymarin exerts anti-renal toxicity by attenuating renal oxidative damage, preserving renal function, and enhancing renal architectures [18].

## Conclusion

ZnONPs are synthesized from the terrestrial *Bacillus paramycoides* strain (MCCC 1A04098 locally isolated) and recorded in vitro antioxidant and antibacterial activities against *S*. *aureus*, *E*. *coli*, and *B*. *cereus* and *C*. *albicans*. It also showed cytotoxic and apoptotic effects on breast cancer and hepatic cancer cell lines. ZnONPs exerted in vivo promising role against hepatorenal injuries in rats.

## Data Availability

The datasets generated during the current study are available at: https://www.ncbi.nlm.nih.gov/nuccore/MT102429 (Accession number: MT102429). All data in this manuscript are available upon request.
